# Hospital volume allocation: integrating decision maker and patient perspectives

**DOI:** 10.1007/s10729-021-09586-w

**Published:** 2021-10-28

**Authors:** Elisabetta Listorti, Arianna Alfieri, Erica Pastore

**Affiliations:** 1 Centre for Research in Health and Social Care Management (CERGAS) SDA Bocconi School of Management, Bocconi University, Milan, Italy; 2 Department of Management and Production Engineering (DIGEP), Politecnico di Torino, Torino, Italy

**Keywords:** Hospital planning, Decision maker, Patients, Volume of activity, Adjusted mortality rate, Volume–outcome association, Operations research

## Abstract

Planning problems in healthcare systems have received greater attention in the last decade, especially because of the concerns recently raised about the scattering of surgical interventions among a wide number of different facilities that can undermine the quality of the outcome due to the *volume-outcome* association. In this paper, an approach to plan the amount of surgical interventions that a facility has to perform to assure a low adjusted mortality rate is proposed. The approach explicitly takes into account the existing interaction among patients’ choices and decision makers’ planning decisions. The first objective of the proposed approach is to find a solution able to reach quality in health outcomes and patients’ adherence. The second objective is to investigate the difference among solutions that are identified as optimal by either only one of the actors’ perspective, i.e., decision makers and patients, or by considering both the perspectives simultaneously. Following these objectives, the proposed approach is applied to a case study on Italian colon cancer interventions performed in 2014. Results confirm a variation in the hospital planned volumes when considering patients’ behaviour together with the policy maker plan, especially due to personal preferences and lack of information about hospital quality.

## Introduction

Planning problems in healthcare systems have received considerable attention in the last decade [33]. Worldwide decision makers’ agenda includes the need for a territorial reorganization of healthcare service provision, and, at the same time, patients advocate for quality and certainty of clinical outcomes [27]. Changes in the composition of the population, in the medical techniques and in the managerial frameworks increase interest in this topic.

Planning problem management is affected by the perspective taken into account. Planning decisions can be addressed through a decision maker perspective, thus adopting a managerial approach. Also, the patient’s point of view can be taken into account by analyzing it with health economics tools. In the literature, two main approaches consider health problems: the managerial approach and the economic approach.

Considering the literature in the managerial field on health planning problems (especially location and allocation problems), such problems have been approached, so far, mostly at the operational and tactical levels, considering pure managerial objectives (security, accessibility, productivity, and so on) [3, 13, 19, 29]. Instead, the literature on health economics, aiming at examining the patient’s perspective, mainly refers to choice models. Research in this field has provided insights on patient and hospital characteristics affecting the patient hospital choice [2, 15, 26, 32, 34, 35]. These models have been mainly used to examine changes in the behavior of patients in response to government policies [37]. However, the same methodology can also be used to enrich health planning decision processes with insights into patients’ preferences [19]. Some studies addressed both the decision maker and the patient perspective together [24, 28], by introducing peculiar aspects. For instance, the preference of patients towards local hospitals rather than health care centers is addressed in [24], while a probability of moving to specific hospitals is studied in [28].

In the healthcare system, concerns have been recently raised related to the scattering of surgical interventions among a wide number of facilities, due to the increasing amount of researches documenting the risk of undermining patients’ health conditions [25]. This phenomenon has been called *volume–outcome association*, and it reports lower volumes of activity (i.e., the number of interventions performed by a facility) being associated to lower clinical outcomes (e.g., higher mortality rates, comorbidity rates, etc.). The volume–outcome association has been first reported in 1979 [22]. In the last decade, a wide literature has further documented the existence of this relationship [6, 7, 11, 14, 16]. The explanation for the observed trend originates from structural factors and professionals’ experience [8, 11, 17, 18, 20]. In Italy, the National Outcome Evaluation Program (Programma Nazionale Esiti, PNE) [6, 7], a project sponsored by the Italian government, documented the occurrence of the volume–outcome association for fourteen procedures using Italian national data, thus confirming the existence of the relation in the recent clinical practice. This issue raised the need for a change in the planning decisions adopted so far [36].

This paper proposes a new tool to support the territorial reconfiguration of healthcare services to guarantee better clinical outcomes. The perspectives of two different stakeholders of the healthcare sector are taken: the decision maker and the patient. Decision makers are in charge of strategic planning decisions concerning both the localization and the size of facilities designated to provide healthcare services. Patients actually make operational planning decisions, when using the healthcare services, by choosing the treating hospital. In the first part of the paper, the single decision processes of decision makers and patients are modelled. The two models drive to i) the optimal volume allocation among hospitals (decision maker point of view); ii) the optimal choice of the facility where to be treated (patient point of view). The objective is to shed some light on the organizational consequences of both decision maker concerns and priorities, and patient preferences.

**Fig. 1:**
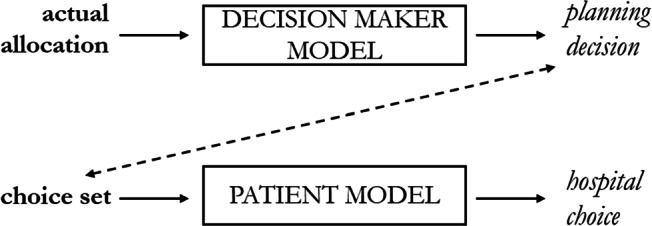
Flow chart of the decision processes of decision makers and patients

As shown in Fig. 1, both decision processes of decision makers and patients have inputs (bold text) and outputs (italic text). As for the decision maker, the input is the actual allocation of interventions among hospitals that, throughout the process, is transformed into a planning decision for a future allocation. As for the patient, the group of alternative hospitals available to be chosen (namely, the *choice set*) is converted into patients’ hospital choice. These two processes are not indipendent. The output of the first process is the input of the second one: patients make their hospital choice based on the choice set that has been determined by decision makers.

As the two perspectives interact with each other, they must be managed together. For this reason, a solution approach is developed to fully integrate patient and decision maker perspectives, by including predictions on patients’ behavior within the decision process of the decision maker. The ultimate objective is to investigate the difference between the optimal solution found by including only one of the actor perspectives, or by including both of them. The aim is to provide a solution that i) reaches quality in health outcomes and ii) ensures patients to operationally realize the planning decision that has been strategically established by the decision maker.

The approach proposed in the paper has three main contributions with respect to the existing literature: i) it considers a strategic decision level; ii) its objective includes only clinical, rather than managerial, outcomes, which remain the main target of all the health care organizations; (iii) it exploits the volume–outcome association in combination with managerial decisions (i.e., volume allocation) and clinical aspects (i.e., consequences on the outcome). The proposed approach is also applied to a case study based on Italian colon surgery patients in 2014. The case study verifies that including different stakeholder perspectives change the territorial planning solution.

The remainder of the paper is organized as follows. Section 2 introduces the problem and how it can be treated considering the single (patient and decision maker) points of view or the integrated perspective. The application of all the solution approaches to a regional case study is reported in Section 3. Section 4 discusses the policy implications for various stakeholders, and Section 5 concludes the paper by summarizing the main results as well as limitations.

## Problem formulation

In this section, the mathematical formulation of the problem is proposed. The addressed problem is the allocation of volumes among the hospitals of a territory, with the objective of achieving better clinical outcomes. The territory includes a geographic region clustered in provinces. In each province, there is a given number of hospitals and patients. A single surgical intervention is addressed in the model, and all the facilities performing that intervention are included in the model. Information about hospitals’ past interventions, i.e., the past performance in terms of clinical outcomes, are assumed to be available to the involved actors. Thanks to epidemiological studies, the total number of patients who require to be operated in a given year is assumed to be known in advance, including their origin.

As discussed in the Introduction, the problem of volume allocation includes different characteristics depending on the adopted perspective (policy maker, patients or both). Hence, two models, each one dealing with the single perspective of each actor, will be presented, together with an algorithm to integrate the decision maker and the patient perspectives.

Before entering into the details of the problem, the main indices of the problem are introduced. Let the index *n* accounts for the province a hospital or a patient refers to, with *N* provinces located in the territory. Index *j* refers to the hospital, and it goes from 1 to a value *J*_*n*_ that depends on province *n*. Similarly, index *i* refers to the patient, and it goes from 1 to a value *I*_*n*_. Each province is allowed to have a different number of facilities *J*_*n*_ and/or patients *I*_*n*_. Index *k* = 1,...,*K* is used to count patients’ personal characteristics.

In the following sections, the different perspectives will be considered, together with the proposed solution approaches.

### Decision maker model

The first perspective is the decision maker’s one, which focuses on how to allocate the annual surgical interventions among the hospitals operating in the same geographical area. The model originates from that presented in [5], which has been tailored to the problem considered in this paper.

When considering the allocation problem, attention must be paid to the factors that can bound the number of interventions allocated to a hospital. To avoid the overload of the structure, or burn-out of the personnel, each hospital ward is considered to have a maximum capacity (in terms of the number of interventions to be performed during the year). At the same time, international guidelines advocate for a minimum annual number of interventions that need to be performed in a hospital in order to guarantee sufficient staff experience and adequate quality levels. These two opposite requirements specify the upper bound and lower bound of the number of operations that can be allocated to a hospital. Moreover, in the considered problem, all the patients that require an intervention are assumed to be able to receive it, in accordance with the universal coverage guaranteed by the Italian healthcare system. Those patients who decide not to be treated within the planning horizon are not included in the model. The hospitals of each province can also treat patients coming from other provinces, but a threshold on the number of interventions that are performed to *foreigner* patients has to be respected for each province (*hospitality threshold*). Eventually, a fixed regional funding imposes an upper bound for the costs of the interventions that can be borne.

Before presenting the mathematical formulation of the problem, its notation is reported.

The decision variables of the model are i) $f_{j_{n}}$ boolean variables, which take value 1 if hospital *j* of province *n* is open, 0 if it is closed; ii) $x_{j_{n}}$ integer variables, indicating the total volume performed by hospital *j* of province *n*.

The parameters of the model are summarized in the following. $cap_{j_{n}}$ is the capacity of hospital *j* of province *n*, whereas *T* identifies the volume threshold suggested by the international guidelines. *δ*_*n*_ identifies the threshold of foreigner patients (in percentage) that can be treated in each province *n*. The marginal cost for each surgical intervention is represented by $c_{j_{n}}$, while *b* is the available regional funding.

As clinical outcome, the *30–day risk–adjusted mortality rate* is selected and used throughout the paper, which represents the main outcome common to all clinical conditions/procedures analyzed in the studies documenting the volume–outcome association [6, 7]. The 30–day risk–adjusted mortality rate is defined as the ratio between the number of patients who died and the total number of patients treated during the same time interval of 30 days following the operation, which is calculated through risk–adjustment techniques. Such risk–adjustment techniques account for the influence of patient characteristics such as age, sex, and comorbidites on the probability of death. Hence, in the proposed model, the adjusted mortality rate of patients being treated in hospital *j* of province *n* is $m(x_{j_{n}})$, where the notation describes its dependence on the volume of performed operations, as stated by the volume–outcome association. The functional form used to model the relationship between the number of interventions and the adjusted mortality rate refers to an Italian work, in order to represent it as close as possible to what occurs in the current clinical practice [6]. Moreover, as in [5], the volume–outcome association is considered in a deterministic setting, i.e., each volume performed is assumed to correspond to a unique adjusted mortality rate. A performance coefficient $v_{j_{n}}$ is assigned to each hospital, which is based on the comparison between the real outcome of the hospital in the year before that planned by the model and the outcome suggested by the mortality curve upon the volume performed in that previous year (i.e., if the model is planning the allocation for year *X*, the coefficient $v_{j_{n}}$ takes into account year *X* − 1). The rationale for the use of the performance coefficient is to tackle those differences in the mortality that are due to variability in surgeons’ talent. In case no information about the previous performance is available, a null performance coefficient is assigned to the hospital, i.e., it is assumed to perform exactly how the volume–outcome curve predicts.

The resulting mathematical model follows.

1 $$ \begin{array}{@{}rcl@{}} min \quad z_{policy} = \sum\limits_{n=1}^{N} \sum\limits_{j=1}^{J_{n}} x_{j_{n}} m(x_{j_{n}}) \\ + \sum\limits_{n=1}^{N} \sum\limits_{j=1}^{J_{n}} x_{j_{n}} v_{j_{n}} dev(x_{j_{n}})\quad \quad \end{array} $$

2 $$ \begin{array}{@{}rcl@{}} \text{s.t.} \quad f_{j_{n}} T \leq x_{j_{n}} \leq f_{j_{n}} cap_{j_{n}} \quad \quad \forall n,j_{n} \end{array} $$

3 $$ \begin{array}{@{}rcl@{}} I_{n} - \sum\limits_{n'=1,n'\neq n}^{N-1} \delta_{n^{\prime}} I_{n^{\prime}} \leq \sum\limits_{j=1}^{J_{n}} x_{j_{n}} \\ \leq I_{n} + \delta_{n} I_{n} \quad \quad \forall n \quad \end{array} $$

4 $$ \begin{array}{@{}rcl@{}} \sum\limits_{n=1}^{N} \sum\limits_{j=1}^{J_{n}} x_{j_{n}} = \sum\limits_{n=1}^{N} I_{n} \end{array} $$

5 $$ \begin{array}{@{}rcl@{}} \sum\limits_{n=1}^{N} \sum\limits_{j=1}^{J_{n}} x_{j_{n}} c_{j_{n}} \leq b \end{array} $$

$$ \begin{array}{@{}rcl@{}} x_{j_{n}} \in Z^{+} , f_{j_{n}} \in \{0,1\} \quad \quad \quad \forall n=1...N, j=1...J_{n} \end{array} $$

The objective function (1) minimizes the total mortality

on the territory, including the hospital performance coefficients. The first term of Eq. 1 is the volume of activity multiplied by the adjusted mortality rate of the hospital, which in turn depends on its performed volume; the second term has the role of increasing/decreasing the mortality depending on the performance coefficient of each hospital, which takes values between -1 (good performance indicator) and + 1 (bad performance indicator). In the sum, the term $dev(x_{j_{n}})$ is the average deviation from the mortality curve corresponding to the volume $x_{j_{n}}$, and it represents the maximum amount of change in mortality that is performance–related. The reader is referred to [5] for the technical details on how both $v_{j_{n}}$ and $dev(x_{j_{n}})$ are calculated.

For each hospital, the operated volume can be either zero (if the hospital is closed, i.e., if $f_{j_{n}} =0$), or (if the hospital is open) it has to be larger than the threshold *T*, but smaller than its capacity (2). Notice that all the hospitals that have a capacity lower than *T* are not considered in the model since they would lead to an infeasible solution. Indeed, this is a further action undertaken to concentrate the regional demand into wards that have sufficient experience.

Constraints (3) consider the province demand satisfaction, by looking at the total volume performed by the hospitals that are in the same province. The inequalities allow hospitals to treat, beyond their provincial population, a *hospitality threshold* of patients coming from different provinces. Vice versa, if patients from province *n* are accepted in the hospitals of other provinces, the hospitals located in province *n* are allowed to treat fewer cases than their provincial population (up to the extreme case where the other provinces treat all the patients from province *n*). The left hand side of equations (3) represents the outflow of patients (namely, the decrease in provincial demand due to patients treated in the hospitals of other provinces), while the right hand side considers the inflow of patients (i.e., patients from other provinces being treated in the hospital). If the hospitality threshold is zero, the hospitals in a province have to treat all the patients living in that province and requiring an intervention.

Eventually, regardless of how the demand is scattered among facilities, all patients must be treated (4). At the same time, the available budget has not to be exceeded (5). The solution of the decision maker model, in terms of volumes assigned to hospitals, is called *strategic solution*.

Summing up, the policy maker model is totally focused on the minimization of total mortality. Constraints are represented by thresholds and capacity issues for volumes, demand satisfaction and allocation of provincial demand among provinces. Hence, the policy maker represents the institutional figure that cares about the whole population health, in terms of both i) universal coverage, ii) quality of the received services. However, in this model, no attention is paid to patients’ answer, i.e., patients are assumed to behave according the volumes strategically planned by the policy maker.

The lack of prediction about patients’ behaviors may lead to differences between the solution planned by the policy maker and the actual volume distribution of patients. Since patients are free to choose the provider to be treated in, they could opt for traveling to any hospital in the region. Also, as no hospital can forbid patients from choosing it, actual changes in the planned volume must be managed. This may cause consequences on hospital capacity stress and, most importantly, on the possible deterioration of clinical quality.

### Patient model

In this section, the decision making process is addressed by the patient, who is concerned about understanding what is the best hospital to be treated in. The patient chooses the hospital according to i) the value perceived from receiving care in a specific hospital (expected utility gained), ii) the comparison among the expected utilities that would be gained if he/she was treated by another available hospital.

The methodology used for this purpose is based on the conditional logit random utility model [23], which is applied to healthcare administrative data (i.e., hospital discharge records), as also done in [9]. As stated in the literature related to choice models, the patient utility can be expressed as the sum of two components, distance and quality [26]. Patients dislike traveling because it is costly and time consuming both for patients and their relatives; hence, the utility decreases if the distance increases. At the same time, patients seek quality care, which increases their utility. Among the quality measures discussed in the literature, the activity volume is used in the paper: the larger number of interventions the hospital performs, the stronger the signal for specialization, experience and thus quality. In the following, the two terms (quality and volume) will be used as synonyms. Patient preferences also vary depending on their personal, social and clinical conditions. For example, disutility caused by distance is generally higher for older patients, possibly because age can act a proxy for frailty and/or for the patient ability to travel [10]. Eventually, the patient decision process consists in a personal trade-off between distance and quality, which strictly depends on the characteristics of the hospitals available to be chosen, and which ends in the choice of the hospital to be treated.

As in the previous section, a given region with its provinces is considered, each of which contains a given number of hospitals. However, since the patient perspective is taken, all the hospitals in the region are considered part of the patient choice set, meaning that they are assumed to be available to be chosen, regardless of the province in which they are located and the province in which the patient lives.

For each province *n*, *I*_*n*_ identical models, each one referred to a different patient *i*_*n*_ living in province *n*, are needed to represent the decision process of every single patient. For each model, the decision variables $p_{i_{n},j_{n'}}$ represent the probability of patient *i*_*n*_ to choose hospital $j_{n^{\prime }}$ of province $n^{\prime }$. This probability expresses the patient propensity for a hospital.

Factors affecting the patient choice, i.e., distance and quality, are indicated by $d_{i_{n},j_{n^{\prime }}}$ and $x_{j_{n^{\prime }},t-1}$. The parameter $d_{i_{n},j_{n^{\prime }}}$ is the distance between each patient of each province *i*_*n*_, and each hospital of each province $j_{n^{\prime }}$. Instead, $x_{j_{n^{\prime }},t-1}$ still indicates the volume allocated to hospital $j_{n^{\prime }}$ of province $n^{\prime }$, and it is used as a quality measure. Differently from the previous decision maker model, $x_{j_{n^{\prime }}, t-1}$ is a parameter, as here patients do not make any choice about the hospital volumes. Moreover, quality is time–varying, so the time at which it is measured must be specified. In particular, patients are assumed to receive information with a time lag of one year, i.e., if the choice is made at time *t*, quality refers to time *t* − 1 (the year before patient choice). By using the time lag, possible reverse causality (i.e., two–way causal relationship) among choice and hospital quality variable is canceled. In fact, demand at time *t* cannot affect mortality at time *t* − 1 [15].

Some coefficients are used to indicate utility variations caused by changes in hospital characteristics. The coefficient β_*q*_ indicates the increase in utility due to an additional intervention operated in the chosen hospital (i.e., due to the hospital quality), while *α*_*kq*_ is the change in β_*q*_ due to the personal characteristic *k*. To include the information about the *k* personal characteristic of patient *i*_*n*_ from province *n*, the parameters $g_{ki_{n}}$ are used. In particular, five patient characteristics are taken into consideration: age, sex, rurality, type of admission (i.e., urgent or elective), and comorbidity.

The changes in utility caused by the change in distance also have some coefficients. The coefficient β_*d*_ represents the increase in utility caused by one additional kilometer traveled to reach the chosen hospital. The coefficient *α*_*kd*_ represents the change in β_*d*_ that is due to the personal characteristic *k*. All the coefficients values are calculated with data, models and methodology presented in [21], to which the reader is referred for all the technical details. It should be noticed that all the parameters are measured through the use of healthcare administrative data (i.e., hospital discharge records), which provide the potentiality of being accessible by all the involved stakeholders of the healthcare system, independently from the direct contact with individuals. This fact enriches the model with the possibility of being replicated, by tailoring it on the characteristics of a specific group of patients.

Patients do not face any strict constraint as for maximum distance traveled and minimum quality required. Rather, the preference function already includes all the aspects of their decision process.

The patient behavior model is represented by the following equations:

6 $$ \begin{array}{@{}rcl@{}} &&p_{i_{n},j_{n^{\prime}}} = \frac{U_{i_{n},j_{n^{\prime}}}}{{\sum}_{n^{\prime}=1}^{N} {\sum}_{j=1}^{J_{n^{\prime}}} U_{i_{n},j_{n^{\prime}}}} \forall n, i_{n}, n^{\prime},j_{n^{\prime}} \end{array} $$

7 $$ \begin{array}{@{}rcl@{}} &&U_{i_{n},j_{n^{\prime}}} = \left( {\upbeta}_{d} + \sum\limits_{k=1}^{K} \alpha_{kd}g_{ki_{n}}\right) d_{i_{n},j_{n^{\prime}}} + \\ &&{}\left( {\upbeta}_{q} + \sum\limits_{k=1}^{K} \alpha_{kq}g_{ki_{n}}\right) x_{j_{n^{\prime}},t-1} \forall n,i_{n}, n^{\prime},j_{n^{\prime}} \end{array} $$

8 $$ \begin{array}{@{}rcl@{}} &&\sum\limits_{n^{\prime}=1}^{N} \sum\limits_{j=1}^{J_{n^{\prime}}} p_{i_{n},j_{n^{\prime}}} =1 \end{array} $$

9 $$ \begin{array}{@{}rcl@{}} &&p_{i_{n},j_{n^{\prime}}} \in \{0,1\} \forall n=1...N, n^{\prime}=1...N, \\ && i_{n}=1...I_{n}, j_{n^{\prime}} = 1...J_{n^{\prime}} \end{array} $$

The patient function (6) represents patient’s behavior, which is entirely centered on the perceived utility. The probability of patient to choose a specific hospital will increase if the utility perceived by its choice increases and/or the sum of the probabilities of choosing all hospitals decreases.

Equations (7) show that the patient utility is the sum of two main components, related to distance and quality. The magnitude of the impact of distance and quality on the utility is given by the alpha and beta coefficients, and by their interactions with personal characteristics.

Equations (8) bound the sum of the probabilities of going to any hospital to be 1. In this way, the link existing among the chosen hospital and all the available ones is represented: the preference for each hospital affects the final patient choice.

As a result, the probability of each patient of choosing each hospital is obtained. To predict the hospital volume, the following formula can be used:

10 $$ \begin{array}{@{}rcl@{}} x_{j_{n^{\prime}},t} &=& \sum\limits_{n=1}^{N} \sum\limits_{i_{n}=1}^{I_{n}} p_{i_{n},j_{n^{\prime}}}\\ \quad \quad \quad \forall n^{\prime}&=&1...N, j_{n^{\prime}}=1...J_{n^{\prime}} \end{array} $$

As Eq. (10) shows, for each hospital of each province, the total predicted number of patients choosing it is the sum of the probabilities of each patient choosing it. This equation can be also interpreted as assigning to the hospital the volume obtained by multiplying the total number of patients by the average probability of all the patients of choosing it. The solution of the patient model, in terms of assigned hospital volumes, is called *operational solution*.

The patient model considers patients as single individuals making their own choices. Thus, it realistically represents the freedom of choice patients have. Nevertheless, by using the patient model, it can be noticed that patients decide i) independently and ii) based on information referred to the previous year. Because of these two conditions, the real magnitude of the gained utility could be different from the calculated one. The most trivial counterexample would be a patient who, after choosing a hospital because it has operated two hundreds of patients during the previous year, is the only one operated in the year of his/her choice. This patient would indeed fear a decline in the hospital performance due to factors related to surgeons’ skills and the facility preparation. As for surgeons’ experience, even though a high volume performed in the past year indicates quality, the volume performed in the current year acts as additional proof. Even if past surgical episodes remain forever embedded in a surgeon experience, it can be assumed that recent interventions account for the most. Moreover, from a facility point of view, the more recently hospitals have received a similar case, the more prepared they are regarding logistics and organization. All this considered, patients risk facing a disappointing gap among the hospital performance of the previous and the current year.

Moreover, because patients choose independently, they lack a territorial overview of the service provided. Patients do not pay attention to the consequences of their choices in terms of quality guaranteed to the whole population. If there is higher dispersion of demand among facilities, no assurance exists about hospitals performing the threshold number of interventions that allows them to have a sufficient experience. Indeed, the institutional figure of the decision maker is the only one with the role of preserving high quality care for the whole population. To overcome these limitations, an integrated approach is proposed in the following.

### Integrated approach

As previously highlighted, there is a need to consider patient and policy maker perspectives together. In this section, the two perspectives are combined in a unique, integrated model. Since the policy maker makes the last decision on the planning choice, the integrated model is defined with her point of view. However, the aim is to merge the objective of low mortality with a positive patient adherence, so that the integrated model includes the patient answers. In other words, the integrated model represents the point of view of a decision maker who, in order to decide how to allocate the interventions to the hospitals, takes into account also how patients might behave.

In the model, the prediction of patient behavior is included in the decision process of the policy maker. Thus, the assumption of total patient adherence to the decision maker plans is removed. The main idea is to control *ex–ante* how patients would behave with different choice sets, in terms of the probability of choosing a particular hospital within the choice set. To this end, the patient model is used, as it takes into account patients’ preferences. After calculating the probabilities of choice for all patients, they can be exploited to predict the total number of patients who will choose each hospital, i.e., the predicted volume of activity of each hospital. Since the volume is related to the clinical quality, the total mortality is computed by the hospital and, summing up all of them, the total mortality associated with the choice set is provided. Since different choice sets will induce different patient behaviors, it is possible to compare outcomes resulting from different choice sets.

The decision maker perspective is kept in the solution procedure: all patient actions are addressed from a strategic perspective and the clinical quality is the main interest. However, the decision maker also accounts for the patients’ point of view and their single preferences, which allows for a more precise prediction of how patients will answer to multiple choice sets. No consideration about costs is added to the model, to specifically focus on the quality of the healthcare services on a strategic level.

**Fig. 2:**
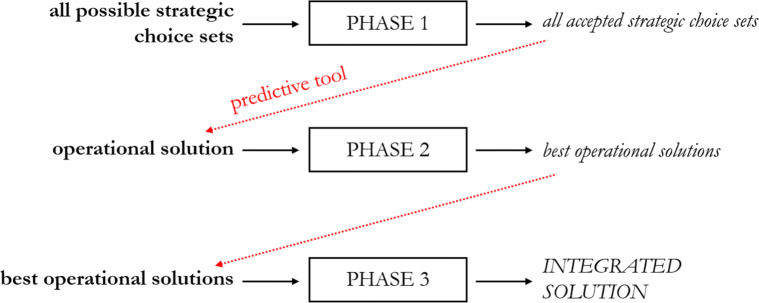
Description of the integrated algorithm

Figure 2 shows the detailed structure of the developed algorithm, which is organized in three phases.

During **Phase 1**, all the available strategic choice sets, in terms of combinations of openings/closures of regional hospitals, are explored. No exploration is made of different volumes of activity that could be attributed to hospitals upon the decision maker model. In fact, patients will act only considering the hospitals present in the configuration, together with their distance and past performance.

All the possible strategic choice sets go through a selection process, where two policy maker constraints are tested. First, the hospitals in the choice set must have operated at least the *T* threshold volume advocated by international guidelines in the previous year. Second, the total capacity of the open hospitals in a province needs to be sufficient to satisfy the provincial request of interventions.

Let *H*_*n*_ be the number of hospitals with a capacity higher than the internationally suggested threshold, and $\tilde r_{n}$ the minimum number of hospitals required to be open together in the provincial choice set (which is calculated by summing up, for each province *n*, the number of *j*_*n*_ hospitals in province *n* with the highest capacities that allow satisfying the whole provincial demand). Then, the number of alternative choice sets in a province is the sum of the combinations that can be formed taking *r*_*n*_ hospitals out of *H*_*n*_, where *r*_*n*_ is an index that can assume minimum values of $\tilde r_{n}$ and maximum values of *H*_*n*_. By combining the provincial choice sets for all the provinces, the regional number of all possible choice sets, *N*_*cs*_, is obtained as follows.

11 $$ \begin{array}{@{}rcl@{}} N_{cs} = \prod\limits_{n=1}^{N} \sum\limits_{r_{n}=\tilde r_{n}}^{H_{n}} \binom{H_{n}}{r_{n}}. \end{array} $$

From the total number *N*_*cs*_, the choice sets that do not respect the second constraint (i.e., the provincial hospitals that do not satisfy the provincial demand) are excluded.

Once all the accepted strategic choice sets are determined, **Phase 2** begins. For each strategic choice set, the patient model is used to calculate the expected probability that each patient will choose each alternative of the choice set. Using the conditional logit in Eq. (10), the average probabilities of the population are assigned to the hospitals as shares of the population that will choose them. In this way, the number of patients that will choose each hospital can be predicted, and this is called *operational solution*. The mortality associated with each operational solution is then calculated. From all the solutions, the three lowest mortality values are computed, and a rule is used to select a subset of operational solutions used as input in Phase 3. All the operational solutions with mortality value equal to the first lowest mortality value are included in the subset. If the second and third lowest mortality values do not exceed the first lowest mortality value of more than *𝜃%*, then all the operational solutions with mortality equal to the second and third lowest values are included in the subset, otherwise they are discharged.

#### Phase 3

consists of a skim process of the selected operational solutions, which can be from a minimum of one (if the second and third lowest mortality values exceed *𝜃%*), up to three, or more in case multiple solutions share the same mortality values. The skim process can be based on a variety of criteria, which should be ultimately defined by who is in charge of the policy implementation, i.e., decision makers and their teams. Three decisional criteria representing key areas widely recognized for their strategic importance [36] are reported here. The three chosen criteria are related to i) structural factors, ii) patients’ preferences, iii) healthcare workforce experience. As for structural factors, priority could be given to make hospitals more prepared to face the demand. In order to do so, the chosen operational solution could be the one that is closer, in terms of volume per hospital, to the strategic solution associated with the strategic choice set (remember that each strategic choice set can be used as an input of the decision maker model, to which an optimal strategic solution corresponds). In fact, hospitals’ organization could realistically be based on this strategic solution, since it is the only information that can be communicated in advance to the hospital managers, enabling them to arrange and adapt layouts and spaces of their facility. The second criterion for the skim process refers to patient utility. If the policy maker is assumed to be interested in maximizing the satisfaction perceived by patients, attention should be paid towards assuring to patients their expected utility levels. Since decisions undertaken by patients are based on past information, policy makers that choose this criterion should minimize the discrepancy between past and actual hospital performance.

Last but not least, the final solution could be based on what will be practically experienced by the healthcare workforce, which is determined by the tactical organization put in place after the choice of the operational solution. For instance, consideration could be given to the presence of university hospitals that, thanks to the support given by students under training, can more easily increase their capacity; or to hospital characteristics in terms of personnel size and shifts, which could be flexible or unable to receive any change.

## A model application to a regional case study

The three solution approaches have been applied to a case study. The case study is related to Piedmont, an Italian region located in the north-west of Italy, which accounts for 4.4 million inhabitants and 8 provinces. In particular, patients that received colon cancer surgery in 2014, which accounted for 1405 people, are considered. The main rationale for the development of the case study is to validate our tool and to show how it works in practice by using the most recent real life data available. The aim is to compare the actual distribution of volumes and outcomes with the ones resulting from the proposed solution approaches, namely the strategic, operational and integrated solutions. The regional context perfectly fits with the prerequisites for the proposed models to be applied, both for the relevance of the illness and the peculiarity of the territorial configuration.

Colon cancer represents, in Italy, the second most frequent oncological pathology: 37100 cases were estimated in 2016, 10% of all the diagnosed cancers [4]. Even if patients who are diagnosed with colon cancer need to be seen regularly, the surgery intervention remains the most invasive aspect of the care process, causing the hospital choice to be a primary concern for patients.

Referring to the volume–outcome association, international guidelines recommend that, for colon cancer surgery, hospitals should provide a minimum of 50 or 70 interventions per year [31]. In Italy, there are concerns that hospitals are undertaking small volumes of colon cancer surgery, the average number of colon surgeries performed by hospitals in 2015 being 34, the median being 19 [1]. In Piedmont, in particular, only 30% of hospitals (15 out of 50) performing colon surgeries in 2014 had a volume larger than 50. The problem of interventions assigned to scattered facilities calls for a policy measure that guarantees the internationally–suggested threshold to be respected.

**Fig. 3:**
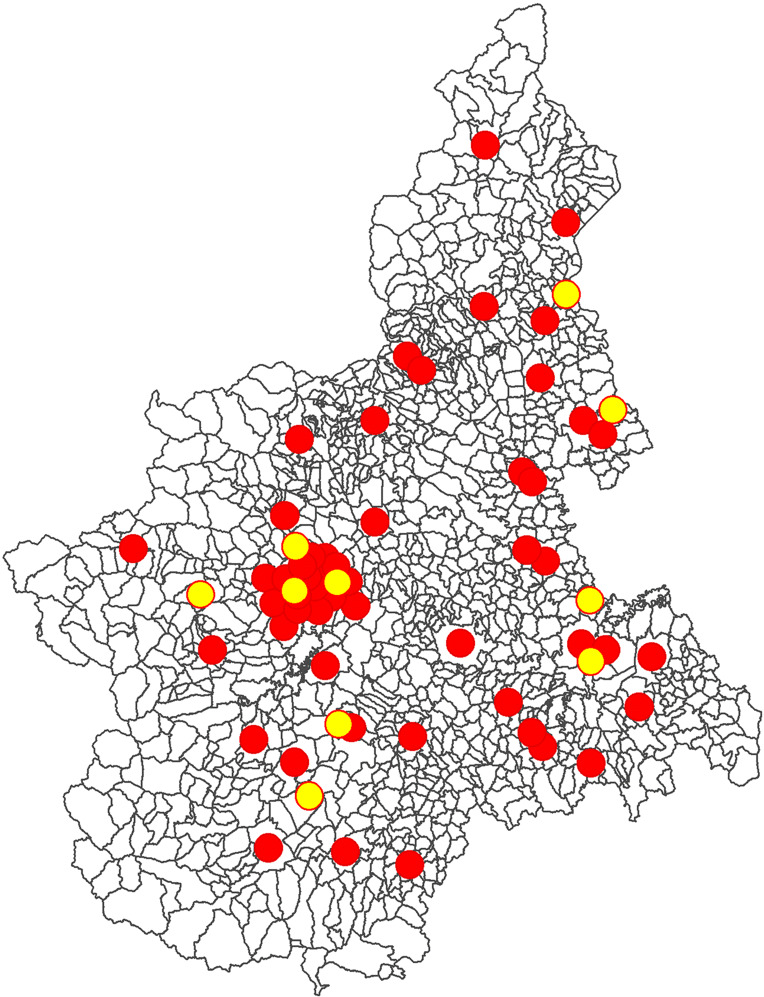
Hospitals location in 2014

**Fig. 4:**
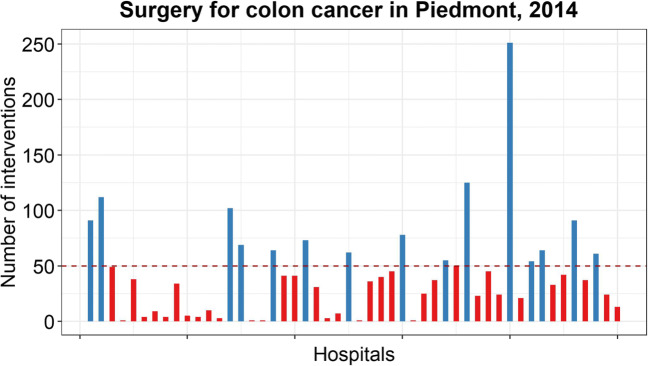
Volume distribution among hospitals, 2014

The actual choice set in terms of hospitals location is graphically shown in Fig. 3 (red dots), while the actual choice in terms of volume allocation is in Fig. 4. It can be noticed that: i) multiple hospitals are located close to each other (Fig. 3); ii) volume allocations resulting from patients’ choices lead to a great disparity in volumes performed by hospitals (Fig. 4). Most importantly, the dispersion of patients among multiple hospitals proves their freedom of choice.

At the end of 2015, a regional administrative order [30] officially identified 24 hospitals as hub centers for colon cancer surgery (which are among both red and blue hospitals shown in Fig. 4). The facilities were chosen based on their experience and specialization in the intervention. The ultimate objective of the policy was to direct all patients towards a restricted group of hospitals to guarantee better clinical outcomes. This policy suggests the willingness of policy makers to change the territorial configuration, thus making our tests of high impact.

In the following, the three proposed approaches are tested in the case study mentioned above. The case study is based on applying the approaches to allocate hospital volumes in terms of colon patients in 2014.

### Decision maker model

The decision maker model includes only the policy maker perspective. The territorial configuration is composed of all the hospitals that operated colon cancer surgery in 2013. In the model, the possibility for the policy maker of closing some of these hospitals is considered. The aim is to evaluate what changes in the volume distribution there would have been if the decision maker model had been applied in 2014.

In order to reduce the computational burden, the special case of each province providing service to its own inhabitants is considered, i.e., the hospitality threshold is set to zero for each province (*δ*_*n*_ = 0∀*n*). By doing so, each province is separately treated, and the complete model is decomposed in *N* = 8 sub–models, one per province. Moreover, no cost constraints (5) are taken into account. Indeed, single hospital cost data are sensible, and they were not publicly available at the time of the analysis.

As for the volume:

1. the hospital capacity is set by assuming that hospitals can perform up to two times the volume performed during the previous year (2013). This assumption is reasonable on a strategic level, where hospitals can be thought to have the possibility to deeply change their internal organization, e.g., hiring more personnel, or using operating rooms for other interventions, etc.
2. the volume lower bound is set such that hospitals are allowed to remain open if they have performed in the previous year *T*/2 interventions. The original lower bound is kept at *T* only for the province of Torino (which is Piedmont capital). In fact, in the territory there is a high concentration of small hospitals, and in some provinces (e.g., AL) the hospitals that satisfy the constraint on the *T* threshold would not have enough capacity to host all their provincial demand.
3. if hospitals performed more than *T* interventions in the previous year (whatever province they belong to), they are forced to remain open. In fact, it is more realistic to close hospitals that are recognized to perform not enough interventions, rather than those that have respected the international guidelines on volumes.

**Fig. 5:**
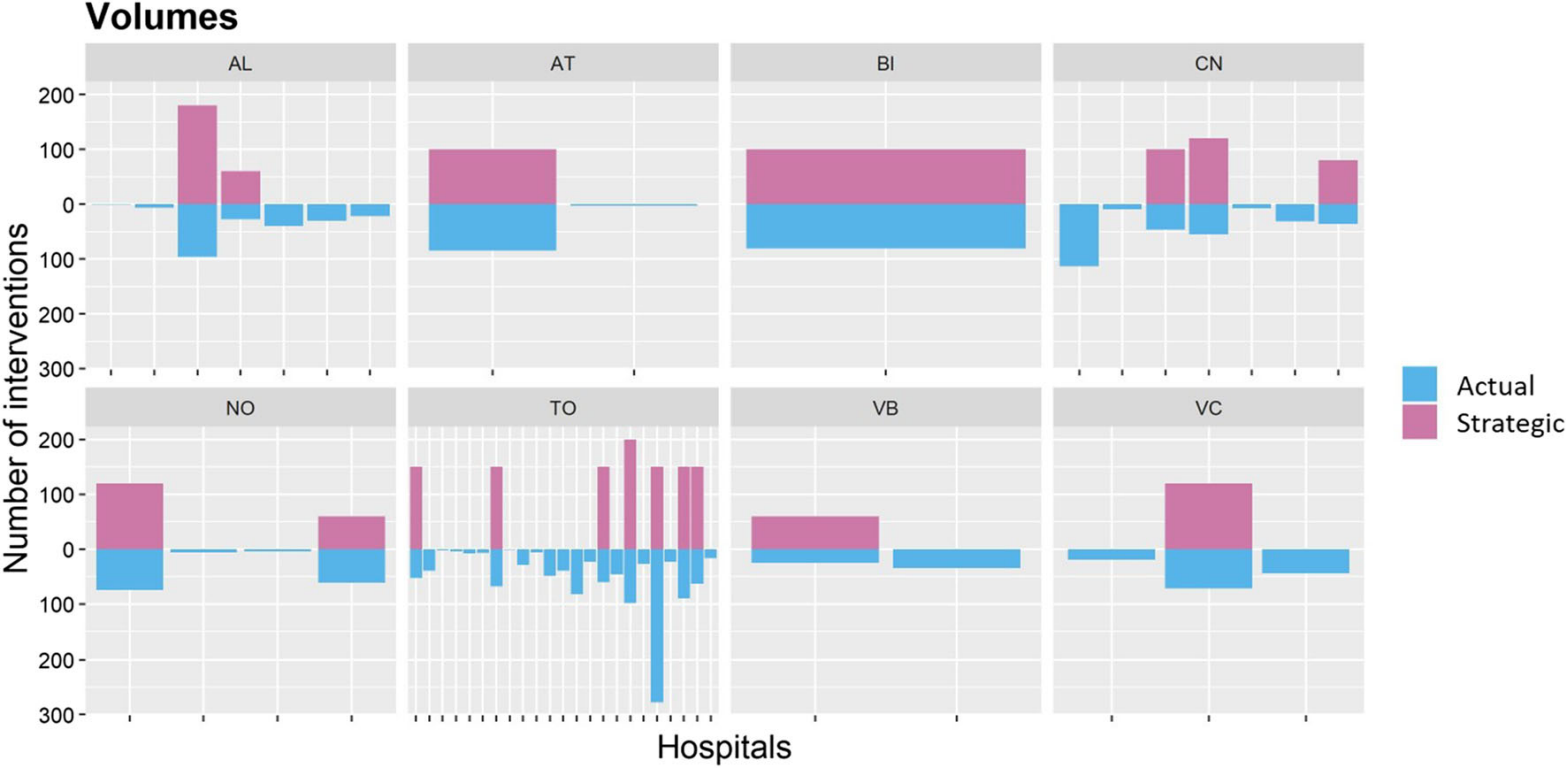
Comparison among volume distributions of actual and strategic solutions

Figure 5 shows the strategic solution resulting from the solution of the *N* decision maker models (purple), and compares it to the actual solution (blue). The two solutions are symmetrically displayed over the horizontal axis; thus, the vertical axis represents the number of interventions of each hospital according to the displayed solution and it takes positive values both upwards and downwards. Each box represents a single province, and each bar represents the number of interventions of a single hospital of that province. The same approach holds for Fig. 7. The suggested configuration is pretty obvious: the minimization of the total mortality drives to the full concentration of interventions in few hospitals. Since the hospitality threshold is set to zero, at least one facility per province must be open, as it happens in the provinces of Asti (AT), Biella (BI), Verbania (VB) and Vercelli (VC). Additional facilities are open in the provinces of Alessandria (AL), Cuneo (VN), Novara (NO) and Torino (TO), where the capacity of the biggest hospital is not sufficient to cover the whole population demand. Because of previous performance information, the model assigns the provincial volume to the hospitals that performed best during 2013.

**Fig. 6:**
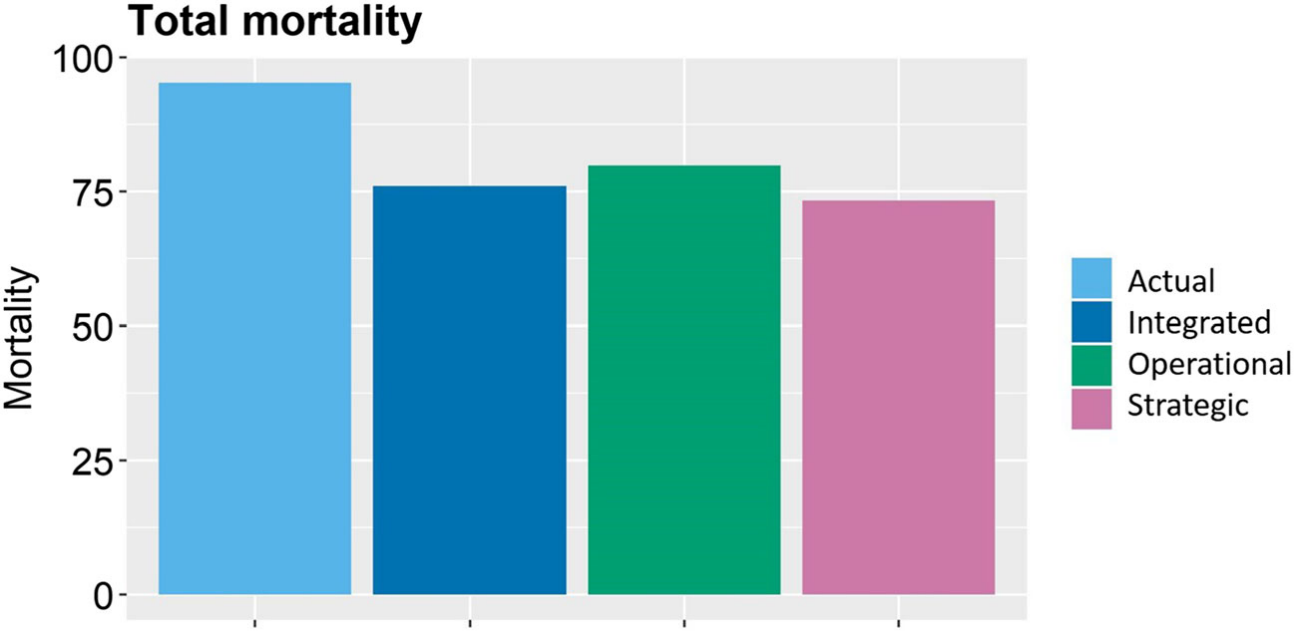
Comparison among outcomes of actual, strategic, operational and integrated solutions

Figure 6 shows the comparison among the regional clinical outcomes (namely, the total mortality) of the actual configuration and the solutions of the three approaches, i.e., the decision maker model, the patient model, the integrated model. Here we focus on comparing the actual and the strategic solutions, and we notice that the strategic solution leads to an improvement of the 23% of mortality, which decreases from 96 to 74.

However, the strategic solution can be different to what would happen in reality with its hospital configuration. Since patients are free to choose any hospital in the regional choice set, their choices could cause changes in the volume distribution (with respect to that predicted from the strategic solution) and, as a consequence, in the clinical outcome.

### Patient model

The patient model can be used to predict patients’ behavior since it calculates, for each patient, the probabilities of choosing each hospital through a conditional logit. The model is applied to Piedmont patients that received colon cancer surgery from 2004 to 2013, to obtain the values for the coefficients β_*d*_, *α*_*kd*_, β_*q*_, and *α*_*kq*_. The coefficients are used in the predictive model, applied to patients that needed elective or emergency colon surgery in 2014.

**Fig. 7:**
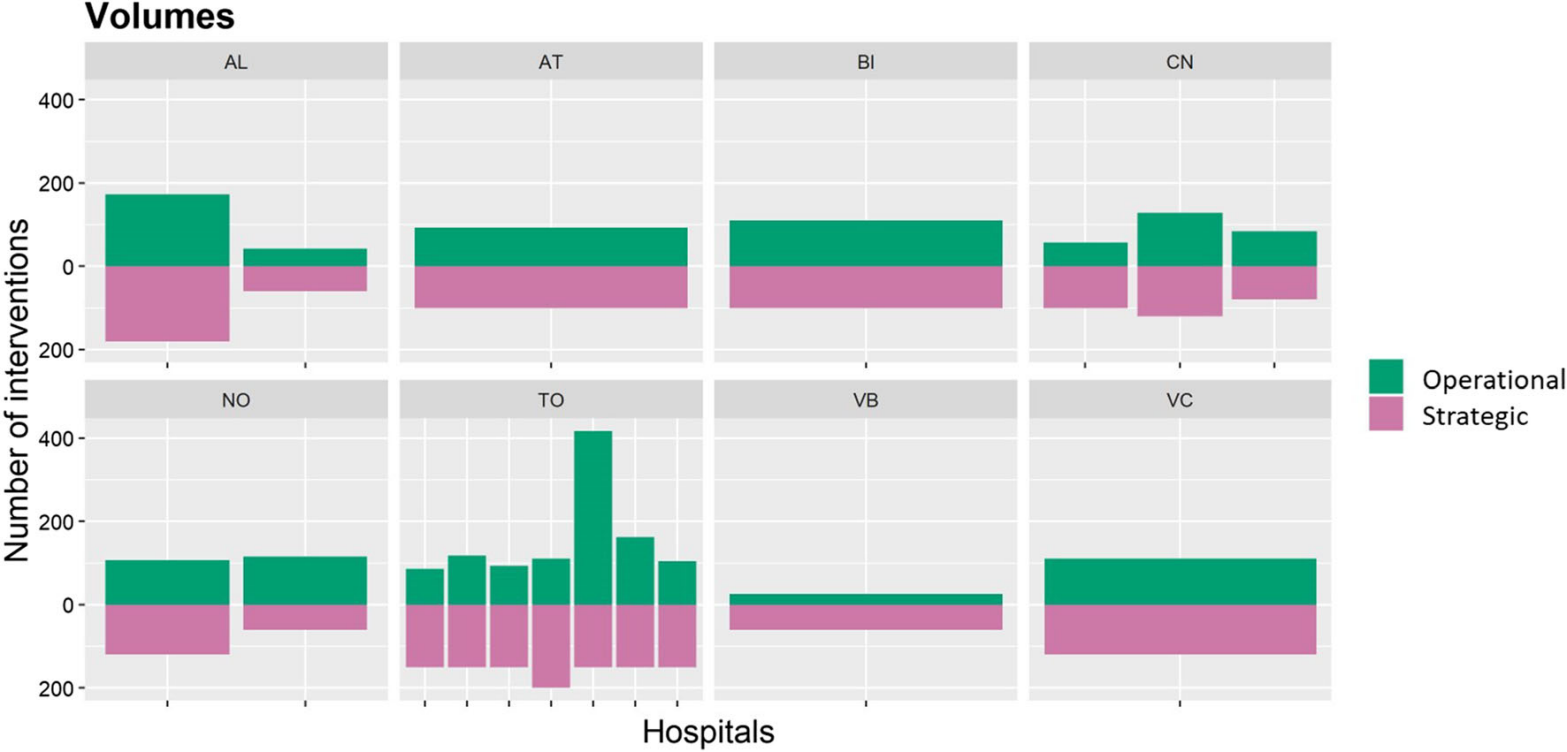
Comparison among outcomes of operational and strategic solutions

The predictive model is applied to the choice set resulted from the strategic solution. The volumes resulting from patient decisions represent the operational solution, which is compared to the strategic solution in Fig. 7. As the decision maker model does not focus on the single patients’ actions, the operational and the strategic solutions cannot be compared at the individual level. For instance, although in Alessandria province the resulting volumes of the two solutions are very similar, it cannot be stated that patients *behave as expected*, because no expectation exists for the decision maker at the single individual level.

The only plan at the individual level that the decision maker proposes is, for each province, that all the patients are treated in their provincial hospitals. With the operational solution, the probability of patients to choose one hospital in their province is on average 17%, while it decreases to an average of 0.9% for a hospital outside of the provincial borders. The average values are computed over all the hospitals of all provinces and all the population. Moreover, if the sample is restricted only to patients that have a unique hospital in their province (i.e., patients from the provinces of Asti, Biella, Verbania and Vercelli), the average probability of choosing the unique open hospital in the province becomes 64%. As a consequence, it can be inferred that these patients, mainly due to the disutility for distance, will be much more prone to choose their closest hospital. On the other hand, the closure of multiple hospitals, by increasing the volumes of the single open hospital, increases the perceived quality of that hospital and then the patient utility.

From Fig. 7, it can be seen that, in the majority of the hospitals, the aggregation of the individual patient choices results in a volume allocation that differs from the one planned by the decision maker. For instance, in the province denoted by TO different bar heights can be noticed by comparing the green (operational solution) and the purple (strategic solution) allocations. In particular, two observations raise concerns. First, the sum of patient choices cause, in 2 hospitals out of 18, the total volume performed to be lower than the internationally suggested threshold of 50 interventions. Second, organizational issues can arise. While the increase/decrease of volume (from 2013 to 2014) required by the decision maker represents a strategic decision that is communicated in advance, the variation of volumes caused by patient choices does not allow facilities to be prepared for it. On average, the difference between volumes, at the hospital level, is 53% of the volume performed the previous year.

As an example, a hospital in Torino (San Giovanni Battista Molinette) would receive by the decision maker model the guideline of performing 150 interventions, which represents 27% of its capacity, thus re–allocating the remaining 73% to other duties and interventions.

However, the following year, due to patients’ choices, it would unexpectedly experience a total demand of 389 interventions. The consequences of this overload and of the organizational changes faced by hospitals can not be easily quantified, but they will likely negatively affect also clinical outcomes because of possible crowding and personnel stress.

By comparing at the aggregated level the operational and the strategic solutions, 35% of patients make a choice that differs from what planned by the policy maker. An additional analysis has been performed by using the patient model. The analysis aims to understand how patient behavior can be influenced by the availability of information on the choice set. Specifically, the patient model is used to calculate the number of patients that, had they known the decision maker plan in terms of volumes and not only of opening/closures, would have made a choice different from the one thought for them by the policy maker. Patient behavior is again predicted by the patient model, but this time applying the hospital characteristics that result from the strategic solution in terms of opening and assigned volumes. Interestingly, in this case, only 17% of patients would have made a different choice. In fact, the difference between 35% and 17% can be associated with the patient knowledge of the overall territorial distribution of volumes. Hence, the policy maker needs to address the problem of making patients fully aware of her regional plan.

All the differences between the strategic and the operational solutions cause a change in the total mortality. Figure 6 shows the total mortality of the operational solution, which is 8.08% higher of the one resulting from the strategic solution.

The graph is interesting for two main reasons. First, it confirms that the free choice of patients can lead to a lower clinical quality in hospitals, if compared to the strategic plan developed by the decision maker. Second, it shows that both the strategic and the operational solutions present a huge improvement if compared to the actual solution. This is mainly due to: i) the decrease of available hospitals, even if patient free choice is allowed, globally increases the volume of interventions, and as a consequence the quality; ii) hospitals available to be chosen have been selected through the performance coefficient, hence they will better perform any volume of activity.

All in all, although the freedom of choice can worsen the outcomes, the decision maker intervention on the choice set, through her planning decision, greatly improves the overall quality. However, an integrated model is essential to guarantee the respect of the quality standard advocated by worldwide researchers [27].

### Integrated approach

The integrated approach is applied to the case study to overcome the difficulties of the communication between patients and decision makers, since the decision maker takes charge of predicting patients’ behaviors and adapting the planning decisions to their answer.

As described in Section 2.3, in Phase 1, all the possible combinations of opening and closures of hospitals are explored. Table 1 shows, by province, the values of *H*_*n*_ (i.e., the number of hospitals with capacity higher than the internationally suggested threshold) and $\tilde r_{n}$ (i.e., the minimum number of hospitals required to be open in the provincial choice set). By summing up the values of *H*_*n*_, out of 53 hospitals that performed colon surgery in Piedmont in 2013, only 26 have enough capacity to perform the threshold number of interventions.

**Table 1:** Parameters *H*_*n*_ and $\tilde r_{n}$ for all provinces in Piedmont

	AL	AT	BI	CN	NO	TO	VB	VC
*H*_*n*_	5	1	1	5	2	8	2	2
$\tilde r_{n}$	2	1	1	2	2	4	1	1

By using Eq. (11), the total number of possible choice sets is computed, and the choice sets with provincial capacity lower than the number of required intervention are excluded. The output of Phase 1 is equal to 41580 strategic choice sets.

**Fig. 8:**
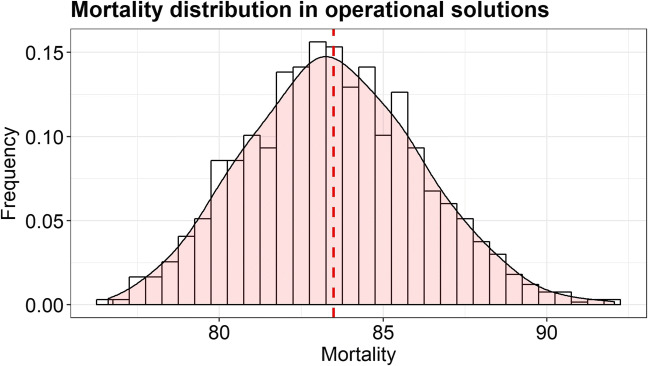
Mortality for operational solutions

At the beginning of Phase 2, the operational solutions are generated and the total mortality is computed for each of them. Figure 8 shows the distribution of mortality values for all the operational solutions. Depending on the strategic choice set that originates the operational solution, hospital volumes resulting from patient choices can widely differ, and, as a consequence, mortality values vary as well.

The coefficient *𝜃* is set to 10%, and, by checking the constraints on the capacity and the threshold *T*, only 3% of operational solutions are selected. Since the second and third lowest mortality values do not exceed the first mortality value of more than 10%, and there are no multiple solutions with the lowest three integer mortality values, the output of Phase 2 is composed of three operational solutions (and, hence, three strategic choice sets), with a mortality ranging from 76 to 77. This gap represents a variation of less than 2%, hence, from a clinical perspective, the group of solutions are interchangeable.

In order to conclude Phase 3, the priority criterion based on structural factors has been chosen. Hence, the decision maker model is run for the three strategic choice sets that resulted as the output of Phase 2. The operational solution whose corresponding strategic solution was closer in terms of volumes assigned to hospitals has been selected, and it is called integrated solution. The integrated solution corresponds to a total mortality of 76.

Observing now the complete picture of Fig. 6, we can clearly state that the best solution in terms of mortality is the strategic solution, which is expected since it is the only solution that comes from the objective function of minimizing the mortality itself. However, as said in the introduction, the objective of the paper is to shed some light on the organizational consequences of both decision maker concerns and priorities, and patient preferences. We do so by examining the magnitude of the existing difference between the values of the first-best solution and all the other solutions.

The first difference emerges between the mortality resulting from the actual and the strategic solutions. The combination of a lower number of hospitals and the closure of facilities with low performance coefficients causes the strategic solution to lead to an improvement of 23% in mortality, which decreases from 96 to 74.

The comparison between the strategic and the operational solutions confirms the results already discussed in Section 3.2.

By looking at the comparison with the integrated solution, interestingly, the mortality of the integrated solution is larger than that of the strategic solution, but lower than that of the operational solution. In fact, the integrated solution represents a choice that is not optimal from the policy maker perspective, but that comes to be optimal when patient answers are considered. Thus, from a theoretical perspective, the first best remains the strategic solution; however, if the aim is to guarantee to the policy maker that the solution will be realistically adopted, the integrated solution becomes the best one.

Two extreme cases can be identified: the actual solution (worst case), and the strategic solution (best case). As the first will be changed anyway, and the second has been said to be effective only on a theoretical level, attention should be paid to the other two solutions. Both the operational and the integrated solutions globally enhance the population health, but the integrated solution reaches better quality in terms of total mortality. Beyond the used mortality indicator, there are multiple managerial factors that make the integrated solution more desirable. In fact, by predicting patient behavior, hospitals can be prepared to face patient answers, even though it is different from the policy maker plan. This represents a huge added value since it avoids overloads of capacity, burn out of medical staff, inefficiency in surgery processes.

The rationale for the development of the case study was to validate the proposed tool and to show that also with real data the solutions involving different stakeholders result in different planning decisions. Also, the case study is useful to show how the proposed tool works in practice. The practical implementation of our model would require the policy maker to choose the parameters following two alternative methods: i) to make predictions on all the parameters that are based on past data; ii) to focus on the robustness of the results originated by a set of scenarios that are reasonable, e.g. suggested by experts’ opinion. A sensitivity analysis would be needed in this last case to analyze the changes in results caused by the changes in the used parameters.

## Policy implications

Behind the findings in terms of key performance indicators, the consequences of the integrated approach must be carefully evaluated. Since it entails a change on the whole planning process, these consequences regard all the stakeholders of the healthcare system. Even though, in this paper, only the policy maker and the patient perspectives are considered, the policy implications will be also discussed for the other stakeholders, i.e., providers, physicians and general practitioners.

- **Policy maker**. The use of an integrated approach translates into a change in her decision process, which brings several advantages. First, the new decision process is built on principles of evidence based medicine (EBM), rather than only on political or managerial concerns. This means that the rationale for the required structural and behavioral changes is supported by the scientific evidence. Therefore, the policy maker is less subjected to critics and scepticism for the reasons that guided the planning choices. Moreover, since the integrated approach includes the prediction of patient behavior, the probability of patient adherence is increased, and, as a consequence, the likelihood of a successful implementation of the planning decisions. Most importantly, given the main objective of the policy maker, i.e., the maximization of the population health conditions, the new decision process ensures to target it.
  However, several criticalities need to be addressed by the policy maker. First, a decision process based on the scientific evidence calls for a high quality of the evidence itself, both in terms of used data and their analysis. For this reason, more attention must be paid to three aspects. (i) The first one is the process of data collection within hospitals. Physicians have to receive specific training to avoid measurement bias due to negligence, lack of understanding, or low priority given to this task of their job. (ii) The second aspect is the commissioning of the analysis of clinical data to expert researchers. (iii) The third aspect is the audit process. The audit process consists in verifying the quality of the data, after the data have been analyzed. As an example, in the PNE system, after the presentation of data online, providers and physicians can raise questions and concerns on the performance scores they have received. The debate helps the researchers to tune the methodologies used for the analysis of data (e.g., to discover the most relevant factors to be considered for the risk-adjustment techniques), and, at the same time, it helps physicians and providers to understand the existing scope for improvement. Handling these three aspects (physicians training, commissioning to data analysts, audit process) will favor the integrated approach to be updated, according to both changes in the practice and in the evidence as well.
  Another controversial aspect that is faced by the policy maker is to guarantee the correct implementation of the changes suggested by the evidence, e.g., the reallocation of volumes among hospitals. In this sense, the creation of incentives acts as a possible support to promote the change, even if attention must be paid to the risk of the so called *gaming*, i.e., opportunistic behaviors on behalf of the involved actors. Examples of incentives are the volume threshold and the pay for performance. Imposing a threshold of interventions that need to be performed is useful to make the providers aware of the importance of the volume–outcome association. Nonetheless, this incentive could lead to weird increases in volumes above the threshold, which could hide over-treated cases that receive the intervention exactly to overcome the threshold.
- **Providers**. The policy built on the use of the volume–outcome association has, as a consequence, the reorganization of the structures that provide healthcare services. Hospitals need to adapt their spaces and staff to the new volumes of activity advocated by the quality standard. Hence, providers are required to think of new management strategies that guarantee efficiency within the new organization. Nevertheless, the increase of volumes can be exploited to reach economies of scale. Moreover, since the concentration of procedures is related to a decrease in mortality, and quality becomes the aspect on which providers are evaluated, the reorganization assures higher quality scores.
- **Physicians**. The change in the allocation of volumes among hospitals has effect also on the organization of the work of the medical staff. This change will surely require attention by the human resources department, in order to avoid burn-out, dissatisfaction or undesirable transfers caused by hospitals closure. However, the most important change that is stimulated by the managerial use of the volume–outcome association is the evaluation of the medical staff based on clinical outcomes. By using discharge records that include the identification of the surgeon, surgeon performance over time can be evaluated. Even if there is the risk for physicians to feel stressed by perceiving the evaluation as a judgment, it can be reckoned that there is a high probability of feeling rewarded for the improvement in their job.
- **General Practitioners (GP)**. The integrated approach fosters the role of the GP as a gatekeeper. In fact, if the integrated approach encourages patients to make their hospital choice, GPs are called to provide their patients the tools to choose. The GP has to guide patients through the use of the published information on quality performance in order to clarify the decision elements that need to be evaluated by the patients themselves.
- **Patients**. The whole integrated approach, together with the importance given to the volume–outcome association, has the ultimate objective of improving patient health conditions. Hence, patients represent the category that receives the highest gain from this new approach. More specifically, patients obtain the strengthening of two aspects: quality and freedom of choice. As for quality, they can rely on healthcare services with lower mortality. As for freedom of choice, they are encouraged to choose the hospital they prefer. However, patients need to be aware of this added value, and they also need to be able to take advantage from it. The territorial reconfiguration, from the patient perspective, causes indeed longer distances to be traveled and could cause longer waiting lists. Even though the two increases do not risk endangering the health conditions, patients need to understand the reason behind these changes. The communication of the EBM strategies, together with the assistance of the GP, could help patients to comprehend that the unique priority is their health.

## Conclusions

This paper proposes an integrated approach to address the problem of planning surgical intervention volumes in a territory with the aim of assuring patient adherence to the plan and high quality of the outcomes, measured in terms of adjusted mortality rates.

The work originates from the observation that, given a specific type of surgical practice, the higher the volume of performed interventions, the lower the adjusted mortality rate, as testified by the so–called volume–outcome association.

The proposed approach uses the perspective of the actor most involved in the planning decision, i.e., the decision maker (or policy maker), but it also takes into account the patient perspective. In other words, the decision maker takes her decision considering the best allocation in terms of quality outcome and also considering how patients will react to the decision.

The approach is tested on a real case study related to colon cancer surgery in the Piedmont region in Italy, by considering the patients that had this surgery in 2014.

The results highlight that the integrated approach gives a solution with larger mortality than the pure decision maker solution (the so-called strategic solution). However, the strategic solution unrealistically assume that patients will follow the design plan, thus it has a purely theoretical value. Instead, the integrated approach includes the choices made by patients, and hence, it will be more likely followed by real patients. The prediction of patient behavior can also allow the hospital to face volumes of activity less different from the planned, thus allowing to avoid, or at least reduce, overloading and inefficiency of the surgical processes. Thus, it can be considered to be realistically the best solution to design. Moreover, it has been shown that the solution reached using whatever perspective (decision maker, patients or integrated) improves the current situation in terms of mortality.

All models and results were eventually presented to the Italian PNE research group, which supports Italian healthcare planning decision. The group positively received the proposed contribution as an opportunity to originate managerial decisions from clinical analyses.

The proposed study presents some limitations. First, the paper only focuses on a single type of medical procedure, while hospital planning takes into account all the types of medical procedures treated in the hospital, due to the overall capacity limit of any structure (the number of beds or operating rooms). To extend the proposed approach to multiple procedures, the synergies between different procedures should be addressed. If the considered procedures require the same skills to surgeons, then data (and volume-outcome association curves) could be aggregated, and the model could be applied on this aggregation level. Otherwise, as the required skills would be procedure-specific, data aggregation can not be applied. In this case, the allocation model should be applied to each intervention, and then an integration of results would be needed to propose and develop the planning decision. The second limitation is related to the volume–outcome association, which is used as a deterministic relation while, in reality, it is affected by uncertainty, and the uncertainty is higher for low volumes. Furthermore, no information about costs was included in the models, differently from previous works [12]. Moreover, in the proposed models, patients’ choices are observed through healthcare administrative data; however, patients base their choices on a variety of structural, process and outcome quality indicators. Although the use of administrative data allows the application of the proposed models on every population characteristics, further research could address the issue of including non-administrative data collected from interviews and surveys, for instance.
